# Optical Performance of RayOne EMV and Tecnis Synergy Under Varying Pupil Sizes and Corneal Aberrations

**DOI:** 10.3390/jcm15031095

**Published:** 2026-01-30

**Authors:** Juan J. Miret, Vicente J. Camps, Celia García, Maria T. Caballero, Ana B. Plaza-Puche, Antonio Sempere-Molina, Juan M. Gonzalez-Leal

**Affiliations:** 1 Department of Optics, Pharmacology and Anatomy, University of Alicante, 03690 San Vicente del Raspeig, Spain; jjmiret@ua.es (J.J.M.); ab.plaza@gcloud.ua.es (A.B.P.-P.);; 2 Department of Condensed Matter Physics, Faculty of Sciences, University of Cadiz, 11510 Puerto Real, Spain; juanmaria.gonzalez@uca.es

**Keywords:** premium intraocular lens, RayOne EMV, Tecnis Synergy, spherical aberration, Through Object MTF

## Abstract

**Background/Objectives:** Premium intraocular lenses (IOLs) are increasingly being selected for cataract and refractive lens surgery, but their functional performance depends critically on pupil size and corneal spherical aberration (SA). This study evaluates how these factors modulate the optical behavior of the RayOne EMV and Tecnis Synergy using a profilometry-based Through Object modulation transfer function (TO MTF) analysis. **Methods:** The surface profiles of the RayOne EMV and Tecnis Synergy were measured with a confocal optical profilometer and implemented in pseudophakic eye models via ray tracing. TO MTF at 50 cycles/mm was computed for object vergences from −4.0 D to +2.0 D over entrance pupil diameters from 2.0 to 5.5 mm in three corneal configurations derived from the Liou–Brennan model and ISO recommendations: mean population SA, aberration-free, and a myopic LASIK-like oblate cornea. Simulated optotype images were generated to relate TO MTF values to the expected distant, intermediate, and near visual performances. **Results:** RayOne EMV delivered high-quality distant image performance in all models. Its depth of focus increased only modestly and showed a strong dependence on pupil size. Intermediate and near vision rarely reached clinically acceptable levels. The Tecnis Synergy produced a broad depth-of-field plateau in distant to near visual performance for mean population spherical aberration at a 3.5 mm pupil. However, image quality at 90 cm remained limited. Optical performance worsened with increasing pupil size and positive spherical aberration, particularly under post-myopic LASIK conditions. **Conclusions:** The RayOne EMV behaves predominantly as a distance-oriented design with minimal true presbyopic benefit; the Tecnis Synergy provides a wider range of vision but is highly sensitive to corneal spherical aberration and pupil size, so thorough preoperative evaluation of corneal asphericity and functional pupil diameter is essential for IOL selection and power targeting.

## 1. Introduction

Modern cataract and refractive lens exchange surgery increasingly employs premium intraocular lenses designed to extend depth of focus beyond traditional monofocal optics. Two distinct optical strategies have emerged: enhanced monofocal and diffractive multifocal/extended-range designs. Enhanced monofocal designs use wavefront shaping (particularly spherical aberration manipulation) to create extended depth of focus. Diffractive multifocal/extended-range designs split incoming light into multiple foci [1]. The RayOne EMV (Rayner, Worthing, UK) is an enhanced monofocal design using increased positive spherical aberration in the central zone to elongate the focal range. The Tecnis Synergy (Johnson & Johnson Vision, Inc., Santa Ana, CA, USA) is designed with an advanced diffractive platform combining trifocal and extended depth-of-focus elements with spherical aberration neutralization [2].

Recent optical bench and modeling studies have characterized the performance of the RayOne EMV and Tecnis Synergy using traditional Through Focus MTF (TF MTF) and related metrics. Schmid et al. used an ISO-2 model eye (corneal SA +0.28 µm) and found that the RayOne EMV delivered excellent distant image quality given an aperture of 3 mm, but only a moderate extension of depth of focus; at 4.5 mm, performance degraded significantly [3]. Alarcón et al. confirmed this strong pupil-dependent behavior using white-light bench methods. This study showed that intermediate visual acuity improved with pupil size at the expense of distance acuity quality, resembling a spherical monofocal IOL [4]. Similarly, Can et al. reported that the Tecnis Synergy showed constrained intermediate acuity quality at 3 mm pupil with an aberration-free cornea, although near vision remained acceptable [5]. Yan et al. found that the Tecnis Synergy offered the best near visual acuity across multiple pupil sizes [6].

The Through Object MTF (TO MTF) is an alternative metric that varies object vergence rather than image plane position, yielding systematically narrower depth of focus (DOD) predictions that better match clinical outcomes [7]. TO MTF is directly related to depth of field (DOFi), recognized as the most clinically meaningful descriptor for multifocal and extended depth-of-focus IOL designs [8]. In previous work, profilometry and through-object MTF analysis were used to evaluate five contemporary IOLs, including the Tecnis Synergy, RayOne EMV, Tecnis Eyhance, Mini Well, and Tecnis Symfony, across different corneal models and pupil sizes. That study showed that premium IOLs with distinct optical designs produce clearly different depth-of-focus profiles and image quality patterns, which are strongly modulated by corneal aberrations and pupil diameter. In particular, enhanced monofocal and diffractive multifocal/extended-range designs demonstrated differing trade-offs between range of vision and sensitivity to spherical aberration and pupil size. These findings underscored the importance of incorporating individual corneal optics and functional pupil characteristics into preoperative planning when selecting and targeting premium IOLs [7].

This initial study was extended in a preliminary paper associated with the present work [9]. In the first part of the analysis, the performance of the Tecnis Eyhance and Mini Well intraocular lenses (IOLs) was evaluated using TO MTF simulations with pupil sizes ranging from 2.0 to 5.5 mm and three corneal models: average aberrated, aberration-free, and post-myopic LASIK. For a typical corneal spherical aberration (SA) and pupils ranging from 3.0 to 3.5 mm, the Mini Well maintained functional image quality from far to near, whereas the performance of the Tecnis Eyhance deteriorated beyond intermediate distances. Lower-than-normal SA induced a hyperopic shift, while higher-than-normal SA induced a myopic shift in far focus for both IOLs. The Mini Well demonstrated greater tolerance to SA changes, while Eyhance behaved increasingly like a monofocal lens [9].

The primary objective of this work is to complete the first study with the RayOne EMV IOL and Tecnis Synergy IOL, characterizing their optical performance across the same wide range of pupil sizes and corneal asphericity conditions. Secondary objectives are as follows: (1) to quantify the sensitivity of each IOL to pupil-dependent and corneal-SA-dependent changes in optical quality; (2) to compare the optical behavior of the RayOne EMV and Tecnis Synergy with that of the Mini Well and Tecnis Eyhance, thereby delineating distinct presbyopia-correction paradigms among four premium IOL designs; and (3) to provide ophthalmologists with evidence-based guidance for preoperative characterization and IOL selection in patients with diverse corneal and pupillary profiles. A secondary exploratory analysis evaluates the effect of IOL power adjustment in compensating for SA-induced focus shifts.

## 2. Materials and Methods

### 2.1. Intraocular Lenses

The RayOne EMV (Rayner, Worthing, UK) is an enhanced monofocal/mono-EDOF design IOL using refractive optics. The optic is biconvex with an anterior aspheric surface; the posterior surface may be an aspheric or spherical shape depending on the IOL power. An aspheric shape and blended zones allow better control of the spherical aberration IOL.

The Tecnis Synergy (Johnson & Johnson Vision, Inc., Santa Ana, CA, USA) features a biconvex optic with a diffractive posterior surface and an aspheric anterior surface (designed with a wave-front guided profile) that incorporates the characteristics of EDOF IOLs.

### 2.2. Surface Measurement Method

The three-dimensional surface topography of each intraocular lens was measured using confocal grid structured illumination implemented with a multimode optical profilometer (Zeta-300, Zeta Instruments, San Jose, CA, USA, Model Z 300). The three-dimensional (3D) image of the surface along the diameter of the lens was obtained by confocal grid structured illumination, with a 100× objective. A stack of 400 images was acquired, covering a vertical range of 280 μm around a starting focal distance of the objective, previously set by autofocus. The field of view of a single image was 0.131 × 0.098 mm^2^. The lens was held in a high-precision XY motorized platform, which allowed us to automate the acquisition of topographic maps across the diameter of the lens. Map stitching was performed by the software of the profilometer to display a compound 2D profile representative of the surface of the IOL. A set of 50 maps was needed to complete the diameter. Raw data were refined using custom smoothing routines and algorithms developed in MATLAB R2022a (The MathWorks, Natick, MA, USA). MATLAB was interfaced with Zemax OpticStudio 2023 R1 (Zemax LLC, Kirkland, WA, USA) via an application programming interface (API) to enable ray tracing simulations and optical performance analysis [7].

### 2.3. Pseudophakic Eye Models and Pupil Sizes

Optical performance was evaluated using the Liou–Brennan finite eye model with modifications following ISO 11979-2 recommendations for simultaneous vision intraocular lenses [10,11]. Entrance pupil diameters ranging from 2.0 to 5.5 mm were tested in 0.5 mm increments. The tested entrance-pupil range (2.0–5.5 mm) was selected to span a functional distribution of pupil sizes commonly encountered from photopic to mesopic viewing conditions in clinical practice. Accordingly, we treat pupil diameter as an independent variable rather than assigning each diameter to a single luminance level. This approach allows the reported TO MTF trends to be interpreted as bounds across lighting conditions, acknowledging that each patient’s effective pupil during daily activities depends on illumination, age, and individual pupillary dynamics. Within the optical model, entrance pupil diameters of 3.5 mm and 5.5 mm corresponded to pupil diameters of 3.0 mm and 4.6 mm, respectively, at the intraocular lens plane, reflecting the pupil-scaling relationship in the eye model. Ray tracing was performed using a monochromatic wavelength (546 nm). The IOL was modeled as centered and coaxial with the optical axis, consistent with ISO-style model-eye assumptions.

Three corneal asphericity configurations were established following ISO recommendations and the Liou–Brennan eye model parameters. The first (Model 1) represented a mean aberrated cornea, approximating the average anterior corneal shape in the general population, with an asphericity chosen to produce a primary spherical aberration of +0.26 μm for a 6 mm entrance pupil. The second configuration (Model 2) modeled an aberration-free cornea, designed to isolate IOL performance by eliminating corneal spherical aberration. In this case, the anterior corneal surface was made more prolate (increased negative asphericity) to yield zero primary spherical aberration, a condition also consistent with naturally prolate corneas or corneas after hyperopic LASIK, where a +4.00 D correction typically induces a marked increase in negative asphericity depending on the platform and ablation profile. The third configuration (Model 3) simulated a myopic LASIK cornea, characterized by a more oblate anterior surface, achieved by shifting asphericity toward positive values until Q = +0.3, corresponding to a myopic correction of roughly −4.00 to −5.00 D. Under these conditions, the resulting primary spherical aberration at a 6 mm entrance pupil reached +0.62 μm.

The IOL axial position and reference planes followed Model 1 configuration. The Effective Lens Position (ELP) and Axial Length were adjusted so that the lens would perform optimally in photopic conditions with an object at infinity, thereby maximizing the MTF value (50 L/mm) in the retinal plane.

### 2.4. Through Object MTF Analysis and Visual Performance Simulation

**Figure 1:**
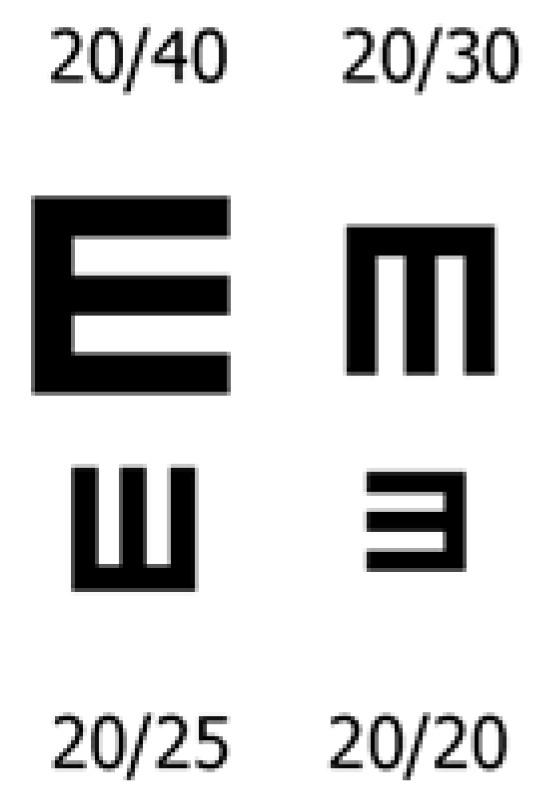
E-Snellen optotype chart and the corresponding simulated visual acuities. The printed “E” is not to scale.

Through Object MTF, curves were calculated at 50 cycles per millimeter (cy/mm) across object vergences ranging from −4.0 D to +2.0 D. The reference plane for TO MTF analysis was positioned at the corneal vertex, while the reference plane for traditional Through Focus MTF was at the intraocular lens plane. The two metrics were mathematically linked via power conversion equations, as previously described [7]. TO MTF performance was validated by generating simulated optotype images spanning expected visual acuities from 20/20 (LogMAR = 0) to 20/40 (LogMAR = 0.3). Simulated images were produced by convolving the point spread function of the combined optical system (cornea and intraocular lens) at the retinal plane with standard E-Snellen optotype patterns (Figure 1). The retinal image plane was defined as the vergence at which the TO MTF at 50 cy/mm for a distant object achieved its maximum value [10].

For Model 1 (mean aberrated cornea) with a 3.5 mm entrance pupil (3.0 mm at the intraocular lens plane), TO MTF curves and simulated optotypes were generated for distance (0 D), intermediate distances (−1.1 D at 90 cm and −1.67 D at 60 cm), and near (−2.5 D at 40 cm) vision across all pupil sizes. For Models 2 and 3, the axial length was held constant relative to Model 1 to represent eyes with identical biometry but only altered corneal asphericity. In these models, only distance-vision optotype images were evaluated to isolate the effects of corneal asphericity on far-vision optical quality.

## 3. Results

### 3.1. RayOne EMV Optical Performance

**Figure 2:**
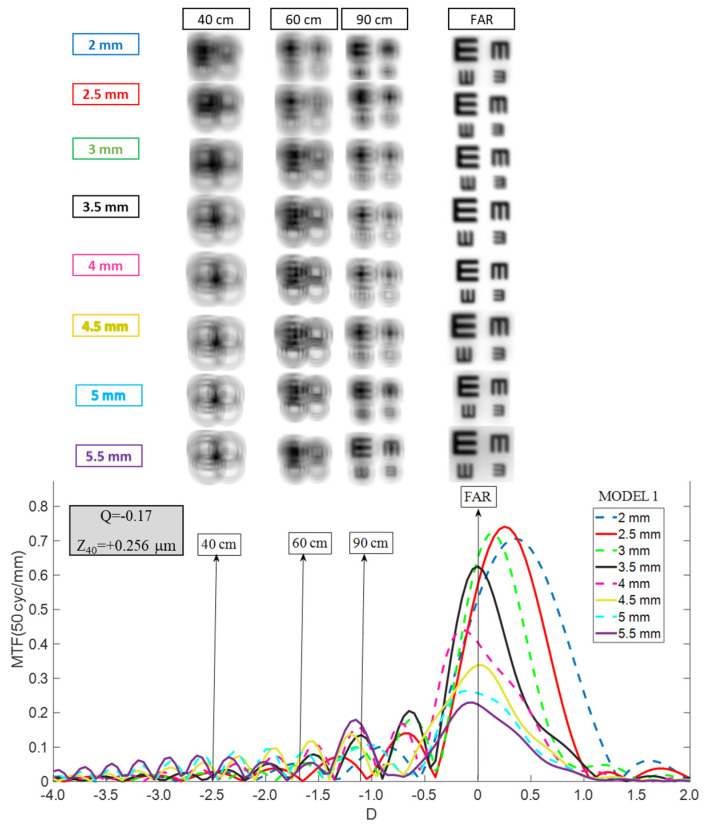
Pupil size dependence of the RayOne TO MTF curve considering Model 1. Simulated optotypes are shown for all pupil sizes and distances.

Model 1 (Cornea With Moderate Aberration): The RayOne EMV demonstrated a moderate depth of focus around the far peak when a 3.5 mm entrance pupil was used (approximately 1.5 D). However, this range of defocus extended further into the hyperopic zone than the myopic zone, contrary to expectation. Consequently, simulated optotype performance (Figure 2) revealed that acceptable image quality was achieved only at far focus; neither intermediate nor near vision reached clinically acceptable levels. The hyperopic shift was more pronounced for smaller pupils, with the mean defocus peak moving to approximately +0.50 D at a 2.0 mm pupil. At larger pupil diameters (4.5–5.5 mm), overall optical quality deteriorated despite a stable distance focus near 0.00 D. Thus, RayOne EMV consistently delivered acceptable image quality only for distance vision, with intermediate-distance performance (90 cm) achievable only at the largest pupil sizes.

**Figure 3:**
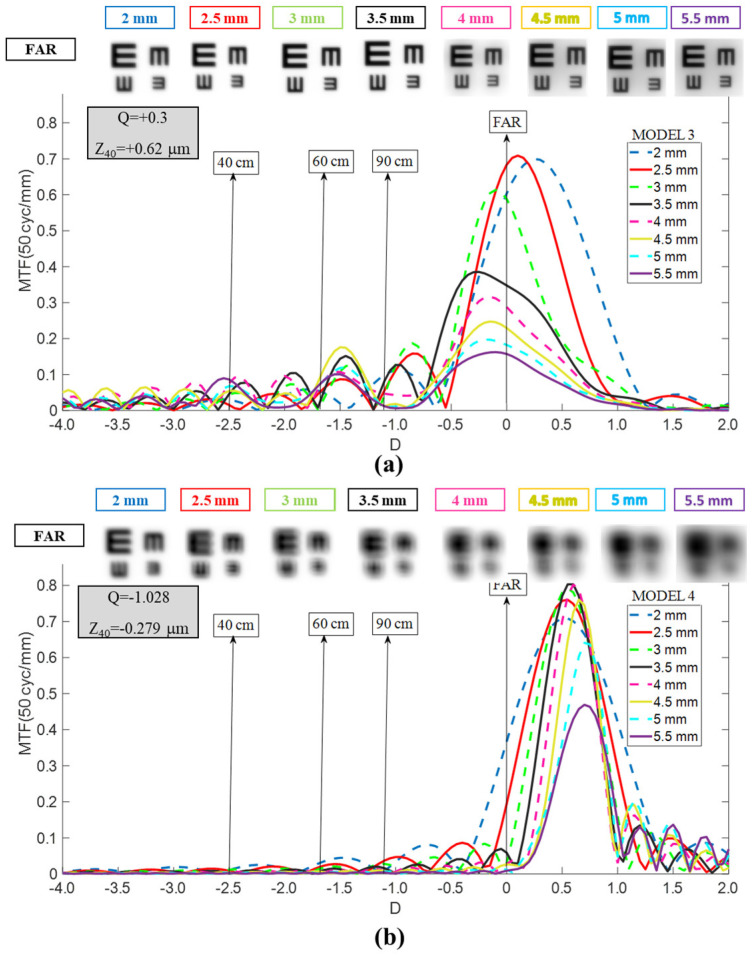
Dependency of the RayOne TO MTF curves on pupil size for Models 2 (**a**) and 3 (**b**). Simulated optotypes for far vision and all pupil sizes are shown.

Model 2 (Aberration-Free Cornea): In the aberration-free corneal model (Figure 3a), TO MTF curves for RayOne EMV were displaced toward hyperopic defocus by approximately +0.10 D to +0.30 D relative to Model 1 across all pupil diameters. Despite the similar shape of the curve, this additional hyperopic displacement further reduced the optical quality at far focus. This exacerbated the limitations observed in Model 1, affecting not only far vision, but also vision at near and intermediate distances.

Model 3 (Myopic LASIK Cornea): In Model 3 (Figure 3b), the RayOne EMV exhibited a consistent myopic shift of approximately −0.10 D to −0.40 D across all pupil sizes. TO MTF magnitude degraded noticeably when pupil diameter exceeded 3 mm. However, far-vision image quality remained clinically acceptable across all tested pupil sizes. As with Model 1, satisfactory optical performance for near or intermediate vision was not achieved.

### 3.2. Tecnis Synergy Optical Performance

**Figure 4:**
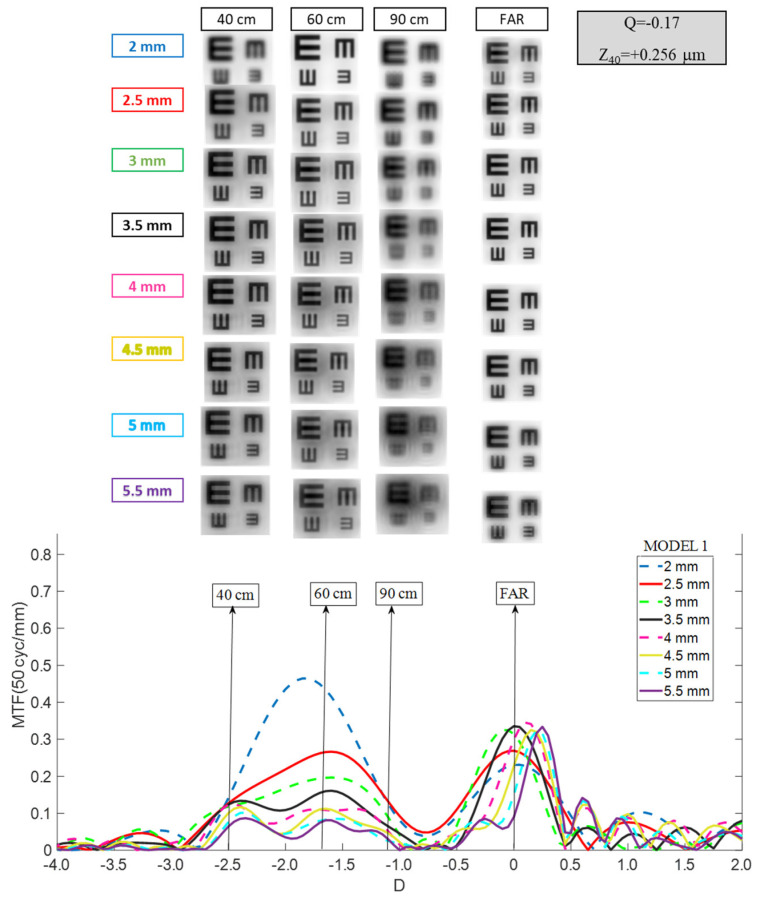
Pupil size dependence of the Tecnis Synergy TO MTF curve considering Model 1. Simulated optotypes are shown for all pupil sizes and distances.

Model 1 (Mean Aberrated Cornea): At a 3.5 mm entrance pupil diameter (Figure 4), the Tecnis Synergy displayed a distinct far-focus peak with an extended plateau spanning intermediate and near vergences. As the entrance pupil diameter increased from 3.5 mm to 5.5 mm, the distance peak gradually shifted toward hyperopic defocus and the intermediate–near plateau progressively narrowed. Conversely, for smaller pupils (2.0–3.0 mm), the distance peak shifted toward myopic defocusing and the plateau broadened, indicating greater depth of focus at intermediate and near distances. Simulated optotype images (Figure 4) confirmed that Tecnis Synergy provided high-quality distance vision and acceptable intermediate vision at 60 cm across all pupil sizes, although contrast degraded as pupils deviated from the 3.5 mm optimum. Visual quality at 90 cm (−1.1 D) remained below the 20/20–20/25 threshold across all pupils. Near vision (40 cm) remained clinically acceptable at all pupil diameters, with reduced contrast compared to distance vision.

**Figure 5:**
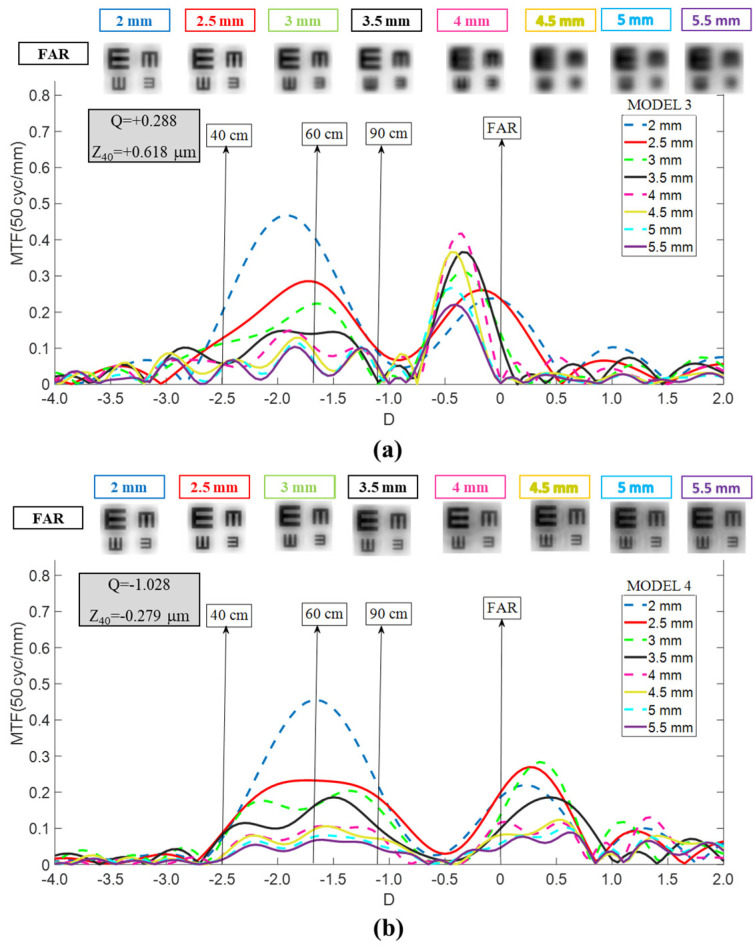
Dependency of the Tecnis Synergy TO MTF curves on pupil size for Models 2 (**a**) and 3 (**b**). Simulated optotypes for far vision and all pupil sizes are shown.

Model 2 (Aberration-Free Cornea): In Model 2 (Figure 5a), TO MTF curves were displaced hyperopically by approximately +0.40 D relative to Model 1. For pupils larger than 3.5 mm, the overall TO MTF decreased substantially, with pronounced reductions at the distance-focus peak, yielding acceptable but lower-contrast far vision. For pupils smaller than 3.5 mm, TO MTF curves remained essentially unchanged relative to Model 1, aside from a slight myopic deflection. A deterioration in optical quality is expected at 40 cm, as the TO-MTF values are generally lower for pupil sizes greater than 3 mm. However, the expected optical quality at 60 cm and 90 cm is the same, with a slight improvement.

Model 3 (Myopic LASIK Cornea): In Model 3 (Figure 5b), the Tecnis Synergy TO MTF curves were displaced myopically by approximately −0.40 D. This shift produced unacceptable degradation of far-vision image quality compared with Model 1, particularly for pupils exceeding 3 mm. For larger pupils (>3.5 mm), additional optical lobes fragmented the intermediate–near plateau, indicating progressive deterioration in overall optical quality. A general narrowing of the near-intermediate plateau is also evident. Consequently, a deterioration in the TO MTF is observed at 40 cm and 90 cm for pupil sizes greater than 3 mm. This behavior will result in a worsening of the optical performance at these distances.

## 4. Discussion

### 4.1. Methodological Approach and Clinical Relevance

This work adhered to ISO 11979-2 standards for simultaneous vision IOL optical assessment and employed Through Object MTF (TO MTF) analysis, which predicts depth of field more accurately than traditional Through Focus MTF (TF MTF). This method yields clinically meaningful outcomes that systematically estimate narrower usable depth-of-field ranges aligned with real-world visual experience [8,10]. The integration of three corneal asphericity models spanned the optical diversity encountered clinically. Model 1 with mean population aberration (Q = −0.18, SA = +0.26 μm), Model 2 with aberration-free optics (Q = −0.57, SA = 0 μm), and Model 3, representing post-myopic-LASIK oblate corneas (Q = +0.3, SA = +0.62 μm), combined with continuous pupil diameter variation (2.0–5.5 mm, corresponding to 3.0–4.6 mm at the IOL plane), provide a comprehensive and realistic representation of the diversity of postoperative refractive laser surgery optical conditions. Because pupil diameter varies with illumination and patient factors, the presented 2.0–5.5 mm analysis should be interpreted as an envelope of expected optical outcomes rather than a single lighting condition. In practice, the most relevant portion of each through-object curve is weighted by the patient’s functional pupil distribution across photopic and mesopic tasks.

Beyond profilometry-based approaches, IOL optical performance has also been investigated through (i) standardized optical-bench testing using ISO model-eye configurations [12] and (ii) computational ray tracing within optical design environments that can incorporate measured or manufacturer-provided surface descriptions [13]. Bench testing provides controlled, standardized comparisons, while ray tracing enables systematic interrogation of design sensitivities (e.g., pupil size, corneal aberration states, and reference-plane definitions) within anatomically motivated eye models. When combined with measured IOL surface topography, ray tracing can serve as a practical bridge between bench metrics and patient-relevant model-eye predictions by providing consistent, repeatable “what-if” analyses under explicitly stated assumptions.

### 4.2. RayOne EMV: Distance-Oriented Design with Limited Presbyopic Extension

Previous optical bench and profilometric studies consistently demonstrate that the RayOne EMV achieves distance image quality comparable to standard monofocal IOLs, with only modest depth-of-focus extension and strong pupil-size dependence. Schmid et al., using ISO standard model eyes with 3 mm and 4.5 mm apertures and monochromatic light (546 nm), reported that the RayOne EMV demonstrated a pronounced MTF peak at 3 mm pupil diameter, with limited depth-of-power extension; however, at 4.5 mm, performance degraded substantially [3]. Alarcón et al., employing the white-light bench methodology with average corneal aberration, similarly found that intermediate simulated visual acuity improved in larger pupils only at the cost of distance MTF reductions, a pattern consistent with spherical monofocal behavior and pronounced pupil dependence [4]. This trade-off is directly confirmed in the present study: functional intermediate vision with the RayOne EMV occurred only at larger pupil diameters (4.50–5.50 mm) and is accompanied by marked degradation of distance optical quality.

The current investigation extends these findings by implementing TO MTF analysis across systematically varied corneal aberration states. In Model 1 (mean aberration, +0.26 μm SA), the RayOne EMV demonstrated approximately 1.50 D of usable depth of focus around distance focus at a 3.0 mm pupil size; this narrowed at larger pupils while exhibiting a hyperopic defocus shift from 0.00 D at 3.5 mm to approximately +0.50 D at 2.0 mm, indicating strong pupil dependence. In Model 2 (aberration-free, 0 μm SA), MTF curves shift hyperopically from +0.25 to +0.70 D, further degrading far-vision optical quality. In contrast, in Model 3 (myopic-LASIK, +0.62 μm SA), a consistent myopic shift of approximately −0.25 D occurred across all pupils. When TO MTF results were compared with prior TF MTF findings, the TO MTF produced systematically narrower and more conservative DOF estimates than the TF MTF, which better align with clinical outcomes. In this framework, RayOne EMV demonstrated quasi-monofocal behavior across all three corneal models, with any apparent DOF extension substantially offset by hyperopic or myopic refocusing induced by corneal aberration changes.

### 4.3. Tecnis Synergy: Hybrid Diffractive Platform with Broad DOF but SA-Sensitive

Optical bench and computational modeling studies describe Tecnis Synergy as an Extended depth of focus multifocal diffractive (EDOF-multifocal diffractive) design combining extended-depth-of-focus and multifocal optical elements. Theoretically this IOL produces a continuous MTF from distant to intermediate and near vision, with characteristic dual-peak or plateau patterns. Can et al., using an ISO standard model eye with population-level corneal aberration and monochromatic light measurements across clinically relevant spatial frequencies, reported that the Tecnis Synergy exhibited two distinguishable foci at a 2.0 mm aperture—one at a distance and a merged intermediate/near peak—while maintaining relatively stable intermediate optical quality as pupils enlarged from 2.0 to 4.5 mm. Notably, optical quality at the intermediate focus degraded substantially at the largest aperture tested (4.5 mm) [5]. Łabuz et al. examined the Tecnis Synergy’s response to corneal aberration by comparing an aberration-neutral model (comparable to our Model 2) with a polychromatic model incorporating population-like SA (comparable to our Model 1). They observed that TF MTF preserved a global trifocal signature with peaks near far, intermediate (~67 cm), and near (~36–40 cm) vergences. At a 2.0 mm pupil under population-level SA conditions, the intermediate and near peaks merged into a plateau. At 3.0 mm, a clear trifocal signature emerged under both conditions; at 4.0–5.0 mm, the far peak dominated while the contributions to intermediate and near vision diminished substantially [14]. These findings aligned with a seminal profilometric analysis by Miret et al., who found TO MTF-based depth of field extending from approximately −2.60 D (38.5 cm) to −1.30 D (76.9 cm) at 3.0 mm IOL-plane pupil with mean corneal SA (+0.27 μm) [7]. The present TO MTF results for Model 1 replicate this pattern: the Tecnis Synergy exhibits a prominent far-focus peak paired with a broad plateau spanning intermediate and near vergences at a 3.5 mm entrance pupil diameter (~3.0 mm at IOL plane), with subtle degradation and lobe emergence as pupils enlarge from 3.5 mm to 5.5 mm, and recognizable quality losses around the intermediate and near vergences. Comparison with historical TF MTF and clinical defocus-curve data—which suggest quasi-trifocal behavior with acceptable acuity down to 33–40 cm—revealed a more conservative pattern with TO MTF: distant and near vision remained acceptable across pupil sizes in the mean-aberrated eye, but intermediate vision at 90 cm (−1.1 D) consistently fell below the 20/20–20/25 visual acuity. Tecnis Synergy demonstrated substantial optical sensitivity to corneal spherical aberration. In Model 2 (aberration-free), TO MTF curves shifted hyperopically by approximately +0.40 D, particularly degrading the distant-vision peak for pupils larger than 3.5 mm, and yielded acceptable but lower-contrast far vision. Conversely, in Model 3 (myopic-LASIK, +0.62 μm SA), a myopic displacement of approximately −0.40 D occurred, producing unacceptable far-vision degradation, especially for pupils exceeding 3.50 mm, where additional optical lobes fragment the intermediate-near plateau and substantially reduce overall contrast.

### 4.4. IOL Power Adjustment and Corneal-SA-Induced Focus Shifts

**Figure 6:**
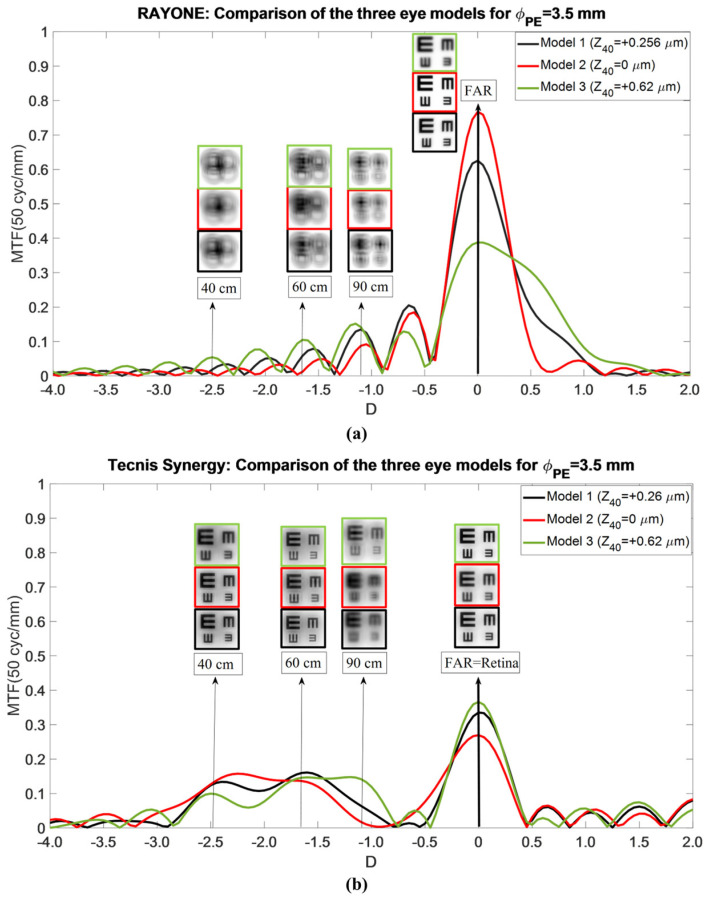
RayOne (**a**) and Tecnis Synergy (**b**) TO MTF curves for the 3.5 mm pupil size and the three models (black color for Model 1, red color for Model 2, and green color for Model 3).

Given that corneal spherical aberration induces measurable myopic or hyperopic shifts in far focus, we explored whether compensatory IOL power adjustments could mitigate these refocus effects across Models 2 and 3 [9]. For the RayOne EMV (Figure 6a), power adjustment yielded limited clinical benefit: distance vision remained acceptable across all models, but near and intermediate vision never achieved clinically acceptable levels regardless of power setting. Far vision degraded progressively as corneal SA became more positive. In contrast, Tecnis Synergy (Figure 6b) demonstrated superior adaptability to power adjustment: far, intermediate (60 cm), and near vision remained acceptable across all models with manageable contrast reduction. Only intermediate vision at 90 cm (−1.1 D) remained poor—with the exception of Model 3 (SA = +0.62 μm), where post-myopic-LASIK conditions unexpectedly supported 20/25-equivalent intermediate acuity. The pupil- and SA-dependent displacement of the distance-focus peak observed in the simulations should be interpreted as a functional refocus that may become clinically meaningful when refractive tolerance is narrow (e.g., premium IOL recipients with high expectations, small functional pupils, or borderline refractive targets). For example, in Model 1, the RayOne EMV distance-peak location shifts toward approximately +0.50 D at a 2.0 mm entrance pupil, which can reduce perceived distance crispness under photopic conditions for patients targeted at emmetropia. Conversely, in routine scenarios, this order of magnitude may fall within typical postoperative refractive tolerances; therefore, the practical impact depends on the patient’s functional pupil distribution and visual priorities. In this context, the power-adjustment analysis (Figure 6) provides a pragmatic framework: designs that maintain acceptable distance image quality across models after modest power targeting (e.g., Tecnis Synergy in our simulations) may allow better compensation of SA-induced refocus than designs whose intermediate/near limitations remain unchanged (RayOne EMV). This differential responsiveness highlights the optical robustness of the Tecnis Synergy EDOF-multifocal diffractive design compared to the RayOne’s refractive strategy, particularly in managing corneal-SA-induced focus shifts.

### 4.5. Four Presbyopia-Correction Paradigms: A Comparative Framework

Integration of the present RayOne EMV and Tecnis Synergy findings with previous TO MTF data for the Mini Well and Tecnis Eyhance [9] reveals four distinct optical paradigms within the premium IOL landscape, each with specific clinical strengths and limitations.

RayOne EMV (Distance-Oriented Monofocal): Delivers maximal distance optical quality (~1.5 D usable depth of focus at 3 mm entrance pupil) with a characteristic hyperopic refocus shift and strong pupil-size dependence in the mean-aberrated eye model. Offers minimal intermediate function restricted to large pupils (only at 60 cm; performance at 90 cm unacceptable). Functional intermediate and near vision were rarely achieved across any corneal model tested. Maintains stable far vision in high-SA post-myopic-LASIK eyes (Model 3) but provides no meaningful presbyopic correction. This simulation could suggest to surgeons that the optimal candidate pool is patients with minimal presbyopic demands who prioritize distance and require good far vision across variable pupil sizes.

Tecnis Eyhance (Robust Distance with Narrow Intermediate Window): Prioritizes robust distance vision (20/20 equivalent across all pupils and corneal models). Offers a narrow, condition-dependent intermediate visual window restricted to optimal pupil diameter (3.0–3.5 mm) with 20/20–20/25 equivalent acuity at 90 cm under mean-aberration conditions. This intermediate window collapses to monofocal-only behavior beyond 4 mm pupils or in high-SA corneas (Models 2 and 3). Demonstrates improved intermediate depth of field in lower-SA models (Model 2), but functionality remains photopic-dependent. The optimal candidate pool could comprise patients with controlled pupil sizes in the photopic/mesopic range (3.0–3.5 mm), normal or reduced corneal SA, and a desire for balanced multifocal vision.

Mini Well (Balanced Multi-Distance with Pupil Optimization): Provides balanced intermediate-to-near-to-distance vision in the optimal pupil window (3.0–3.5 mm entrance pupil) with 20/20 equivalent acuity across multiple distances (far, 60 cm intermediate, and near). Performance degrades outside the optimal pupil window due to lobar fragmentation and reduced contrast at pupil extremes. Demonstrates tolerance to lower-SA corneas (Model 2) but exhibits marked degradation in high-SA post-myopic-LASIK eyes (Model 3). The results suggest that the optimal candidate pool could be patients with controlled pupil sizes in the photopic/mesopic range (3.0–3.5 mm), normal or reduced corneal SA, and a desire for balanced multifocal vision.

Tecnis Synergy (EDOF-multifocal diffractive with Extended Plateau but High SA-Sensitivity): Maintains a broad, relatively pupil-independent MTF near-intermediate vision plateau at the optimal 3.5 mm entrance pupil in mean-aberration conditions, supporting sustained 20/32 or better acuity from distance vision to near vision. However, sacrifices optimal intermediate performance at 90 cm (−1.1 D), which consistently falls below 20/20–20/25 threshold. Exhibits substantial sensitivity to corneal spherical aberration (±0.40 D focus shifts with ±0.36 μm SA variation), with fragmentation of the plateau and unacceptable degradation in post-myopic-LASIK models of large pupils, with a deterioration of near (at 40 cm) and intermediate (at 90 cm) vision quality. Provides an extended range of usable vision at the cost of marked dependence on preoperative corneal profiling and functional pupil prediction/ Consequently, the optimal candidate pool may comprise motivated patients with normal or slightly reduced corneal SA and stable mesopic pupil sizes. These patients should have realistic expectations regarding the 90 cm intermediate distance and prioritize an overall range of vision over intermediate-distance specialization.

To facilitate clinical translation of these results, Table 1 summarizes practical selection and counseling considerations as a function of corneal SA state (Models 1–3) and functional pupil size within the tested 2.0–5.5 mm range.

### 4.6. Limitations

This study is based on model-eye simulations intended to isolate and interpret the sensitivity of premium IOL designs to pupil diameter and corneal spherical aberration. Accordingly, corneal optics were represented through three controlled asphericity/SA conditions rather than a full distribution of higher-order aberrations, and ocular dynamics such as accommodation microfluctuations or neural adaptation were not modeled. The optical analysis also depends on the illumination assumptions used for ray tracing (monochromatic vs. polychromatic) and on idealized alignment (centered, coaxial IOL without tilt/decentration unless otherwise stated). These simplifications support mechanistic comparison across designs but may limit direct generalization to individual eyes with complex HOA patterns, pupil decentration, or clinically relevant IOL misalignment.

## 5. Conclusions

The RayOne EMV and Tecnis Synergy lenses exhibited distinct and model-dependent behaviors across the three corneal conditions considered, which are central to interpreting their presbyopia-correction potential. In the mean-aberrated model (Model 1, population-like positive SA), the RayOne EMV showed a moderate depth of focus of roughly 1.5 D for distance focus for a 3.0 mm pupil, but this extension occurred mainly toward hyperopic vergences and did not translate into clinically acceptable intermediate or near performance in the simulated optotypes. When moving to the aberration-free model (Model 2), the RayOne TO MTF curves shifted further into hyperopic defocus by about 0.10–0.30 D, reducing peak distance MTF and worsening the already limited intermediate and near vision quality, so that the lens behaved even more clearly as a strictly distance-oriented optic. In the post-myopic LASIK-like model (Model 3, high positive SA), the RayOne experienced a myopic shift of approximately 0.10–0.40 D, but far-vision quality remained acceptable across pupils; nevertheless, intermediate and near performance continued to be poor, indicating that corneal-induced refocus does not convert this design into a true presbyopia-correcting solution.

From a clinical standpoint, the small-pupil hyperopic displacement (on the order of a half-diopter in the present simulations) may be relevant for patients with predominantly photopic small pupils and tight distance targets, whereas in many routine cases it may be comparable to expected refractive tolerances; thus, incorporating functional pupil size into preoperative planning can improve power targeting and patient counseling.

The Tecnis Synergy showed a different pattern of dependence in the same eye models. In the mean-aberrated model, it produced a prominent distance peak associated with a broad plateau covering intermediate and near vergences, given a 3.5 mm entrance pupil diameter, supporting good simulated distance and near vision with limited performance only at 90 cm. In the aberration-free model, the entire TO MTF profile shifted hyperopically by roughly 0.40 D, leading to a reduction in distance contrast in pupils larger than 3.5 mm and a subtle reshaping of the intermediate–near plateau, so that far vision became lower-quality while the expected near and intermediate quality depended more critically on precise refraction. In the post-myopic LASIK-like model, the Tecnis Synergy experienced a comparable myopic displacement of about 0.40 D, but here, the impact was more disruptive: for pupils above 3.0–3.5 mm, additional optical lobes appeared and the plateau fragmented, causing unacceptable degradation of far-vision image quality and a narrowing and destabilization of the intermediate–near range. Altogether, these dependencies indicate that the RayOne EMV preserves a relatively robust but essentially monofocal distance performance across eye models, whereas the Tecnis Synergy offers a wider functional range but is highly contingent on corneal spherical aberration and pupil size, with its best behavior confined to eyes with population-like or mildly reduced SA and controlled mesopic pupils.

These model-dependent behaviors of the RayOne EMV and Tecnis Synergy help contextualize the previously characterized Mini Well and Tecnis Eyhance designs within a unified presbyopia-correction framework. When these findings are integrated with earlier TO MTF simulations, Mini Well emerges as a design that offers balanced far, intermediate, and near vision within a restricted photopic–mesopic pupil window (around 3.0–3.5 mm), and it shows relatively good tolerance to reduced or normal spherical aberration, while exhibiting clear vulnerability in high-SA, post-myopic LASIK eyes, where its multi-distance plateau fragments and contrast deteriorates.

Tecnis Eyhance, by contrast, maintains robust distance vision across a broad range of pupils and corneal profiles, but it provides only a narrow, condition-dependent intermediate window that requires near-optimal pupil size and corneal asphericity and collapses toward essentially monofocal behavior when encountering large pupils or elevated positive spherical aberration. In combination with the present RayOne EMV and Tecnis Synergy results, these earlier observations delineate a continuum ranging from distance-prioritizing designs with limited presbyopic benefit (the RayOne EMV and Tecnis Eyhance in non-ideal conditions) to lenses that deliver broader depth of field at the cost of stricter requirements on corneal optics and pupil control (the Mini Well and especially the Tecnis Synergy), emphasizing that the relative advantages of each platform depend critically on the specific corneal model and functional pupil characteristics of the eye under consideration.

Overall, the four lenses exemplify distinct presbyopia-correction paradigms—distance-oriented quasi-monofocal (RayOne EMV), distance-prioritizing with limited intermediate (Tecnis Eyhance), balanced multi-distance within an optimal pupil range (Mini Well), and EDOF–multifocal diffractive with an extended but SA-sensitive plateau (Tecnis Synergy)—whose performance is strongly modulated by corneal asphericity and pupil diameter. These results underscore that premium IOL selection should not rely solely on target refraction but must also incorporate individualized assessment of corneal spherical aberration and functional pupil size, using them to guide lens choice, power targeting, and patient counseling regarding the expected trade-offs between far, intermediate, and near visual performance.

## Figures and Tables

**Table 1:** Summary of practical decision aid linking corneal SA state and functional pupil size to IOL-paradigm selection and counseling on the four IOLs [7].

Patient Profile Anchor (from This Study)	Functional Pupil Tendency (Entrance Pupil)	Preferred Paradigm(s) (from Section 4.5)	Practical Expectation/Caution (from Present Results)
Low/neutral corneal SA (Model 2; SA ≈ 0 µm at 6 mm)	Small-to-medium (≈2.0–3.5 mm)	Distance-prioritizing designs (RayOne EMV; Tecnis Eyhance) and selected extended-range options	RayOne EMV shows hyperopic displacement vs. Model 1 and limited intermediate/near vision; counsel as distance-oriented. Synergy may show hyperopic displacement (~+0.40 D vs. Model 1) and reduced distance contrast at larger pupils.
Population-like positive SA (Model 1; SA ≈ +0.26 µm at 6 mm)	Medium (≈3.0–4.0 mm; includes 3.5 mm “optimum” in this study)	EDOF–multifocal diffractive (Tecnis Synergy) for range; balanced multi-distance (Mini Well) within optimal pupil window	Tecnis Synergy shows broad plateau from distant to near vision at 3.5 mm; 90 cm remains comparatively limited for all pupil sizes. Mini Well and Tecnis Eyhance performance depend on remaining within the optimal pupil window.
High positive SA/post-myopic LASIK-like (Model 3; SA ≈ +0.62 µm at 6 mm)	Medium-to-large (≥3.0–5.5 mm)	Distance-prioritizing choices may be safer (RayOne EMV; Tecnis Eyhance depending on pupil)	Tecnis Synergy shows myopic displacement (~−0.40 D) with unacceptable far-vision degradation for pupils >3.0–3.5 mm and plateau fragmentation; counsel carefully. RayOne EMV keeps acceptable far vision but does not provide meaningful near/intermediate vision.
Strong presbyopic demands + acceptance of multifocal trade-offs	Any (weight by patient’s real-world pupil distribution)	Tecnis Synergy/Mini Well (if cornea/pupil compatible)	Emphasize that range of vision depends on corneal SA and pupil; use this table to match expectations and discuss the 90 cm intermediate limitation noted for Tecnis Synergy under several conditions.
Minimal presbyopic demands; distance priority	Any	RayOne EMV/Tecnis Eyhance paradigm	Counsel that RayOne EMV behaves quasi-monofocally with limited true presbyopic benefit; intermediate gains may appear only for larger pupils and may reduce distance vision quality.

## Data Availability

The data supporting the findings of this study are available from the corresponding author upon reasonable request.

## Abbreviations

The following abbreviations are used in this manuscript:

IOL	Intraocular Lens
SA	Spherical Aberration
EDOF	Extended Depth of Focus
MTF	Modulation Transfer Function
TO MTF	Through Object Modulation Transfer Function
TF MTF	Trough Focus Modulation Transfer Function
DOF	Depth of Focus
DOFi	Depth of Field
